# BMSCs improve TNBS-induced colitis in rats by inducing Treg differentiation by expressing PD-L1

**DOI:** 10.1007/s10529-022-03307-1

**Published:** 2022-10-20

**Authors:** Fei Gao, Dandan Cui, Dongmei Zuo, Zhexing Shou, Jia Yang, Ting Yu, Yujin Liu, Si Chu, Feng Zhu, Chunzhu Wei

**Affiliations:** Department of Integrated Traditional Chinese and Western Medicine, Union Hospital, Tongji Medical College, Huazhong University of Science and Technology, Wuhan, 430022 China

**Keywords:** Bone marrow-derived mesenchymal stem cells, Inflammatory bowel disease, Tregs, PD-L1

## Abstract

**Objectives:**

Bone marrow-derived mesenchymal stem cells (BMSCs) show promise in treating inflammatory bowel disease. We tested if BMSCs improve Trinitro-benzene-sulfonic acid (TNBS)-induced colitis by inducing Treg differentiation by modulating programmed cell death 1 ligand 1(PD-L1).

**Results:**

BMSCs were isolated and transfected with PD-L1 siRNA. Sprague–Dawley rats were randomly divided into 4 groups: normal, model, BMSC control, and PD-L1 siRNA BMSC. Colitis was induced by TNBS, except in the normal group. On d4, the BMSC control and PD-L1 siRNA BMSC groups were intravenously injected with BMSCs at a dose of 5 × 10^6^ cells in phosphate-buffered saline (PBS; volume matched). BMSCs were later verified to have reached the colon tissue. BMSC control showed significantly better clinical symptoms and reduced histopathological colitis severity; PD-L1 siRNA BMSC group showed no difference. PD-L1 siRNA reduced: spleen and mesenteric lymph node Tregs, PD-L1, interleukin-10 (IL10), phosphate and tension homology deleted on chromosome ten (PTEN); colon p-Akt and p-mTOR were increased.

**Conclusions:**

We found that BMSCs can induce Treg differentiation by inhibiting the Akt/mTOR pathway via PD-L1; this significantly improved symptoms and pathology in our ulcerative colitis rat models.

## Introduction

Ulcerative colitis (UC) is an inflammatory bowel disease (IBD) characterized by chronic inflammation and ulceration in the colon and rectum; typical symptoms include recurrent abdominal pain and bloody mucous diarrhea. Diagnosis is via colonoscopy and pathological examination. UC, previously considered a low-risk lesion, has been gradually increasing in incidence in Asia, and is now regarded as an significant risk factor for colorectal cancer Bopanna et al. 2017, Ng et al. 2016). The etiology and pathogenesis of UC are still incompletely understood but most researchers think that UC onset is associated with environmental factors, genetic susceptibility, and immune dysfunction. Compelling data reveal that the most critical factor contributing to the development of UC involves defects in intestinal mucosal immunity (Ungaro et al. 2017). The central problem of such intestinal immune dysfunction can be characterized as an imbalance between pro-inflammatory and anti-inflammatory cells, which mainly involves the T helper subset Th17 cells and regulatory T cells (Treg). Tregs, important anti-inflammatory cells, play a pivotal role in attenuating UC by activating Forkhead Box P3 (FOXP3) and secreting the anti-inflammatory cytokine IL-10 (Schmitt et al. 2020; Xu et al. 2017).

Bone marrow-derived mesenchymal stem cells, a type of non-hematopoietic stem cell that exist in the bone marrow, are widely used to study stem cell-based therapeutics. Due to their multi-directional differentiation and high proliferation potential, BMSCs can be induced directly into neural cells, osteoblasts, fat cells, etc. given specific culture conditions (Grégoire et al. 2017; Liu et al. 2019). Although they only account for 0.001–0.01% of bone marrow monocytes, BMSCs can be expanded over 1 million times or 6 generations in vitro (El Agha et al. 2017). Increasingly, studies have reported that BMSCs show great immunosuppressive potential and can be used to treat inflammation-mediated diseases such as IBD (Zhou et al. 2020). In our previous study, we demonstrated that BMSCs can relieve TNBS-induced colitis by increasing the percentage of circulating Tregs in rats; as such, we believe that BMSCs alleviate colitis by promoting Treg differentiation. However, the regulatory mechanisms linking BMSCs and Tregs remain to be elucidated (Nan et al. 2018; Zuo et al. 2013).

PD-L1(also called CD279 or B7-H1), a ligand of programmed cell death-1 (PD-1), is a member of the CD28/B7 superfamily and is expressed widely (Ishida et al. 1992; Sun et al. 2018). The PD-1/PD-L1 co-stimulatory signal is mainly involved in the central and peripheral immune tolerance of CD4^+^ T cells and can regulate the balance of effector T cells and Tregs in the progression of multistage autoimmune diseases (Sun et al. 2018; Zhang et al. 2018). Some studies show that PD-L1 is the most important factor in inducing naïve T cell differentiation into Tregs, which occurs via the inhibition of the phosphatidylinositol-3-kinase/mammalian target of rapamycin (Akt/mTOR) signaling pathway (Davies et al. 2017a, b). At the same time, BMSCs are reported to inhibit the activation and proliferation of CD4^+^ T cells via the PD-1/PD-L1 pathway (Chen et al. 2018). Based on these findings, we hypothesized that BMSCs alleviate the rat colitis model by inducing Tregs differentiation via PD-L1.

## Materials and methods

### Isolation, culture, and identification of rat BMSCs

BMSCs were isolated from 3 week-old healthy male SD rats as described previously. BMSCs were collected from femurs and tibias by flushing medullary cavities and then were cultured in flasks using low-glucose complete cell culture medium consisting of α-minimum essential medium (α-MEM; Gibco, Invitrogen Corp., Grand Island, NY, USA) containing 10% fetal bovine serum (FBS; Gibco, Invitrogen Corp.). The cells were incubated in 5% CO_2_ at 37 ℃. Non-adherent cells were removed at each media change; adherent cells were collected by using 0.25% trypsin solution (Gibco, Invitrogen Corp.) during passaging. BMSCs from the second passage (P2) were used for phenotyping and all following experiments. For flow cytometry phenotyping, we utilized anti-rat CD-29, CD-90, CD-45, CD-11b antibodiesy (BioLegend, San Diego, CA, USA). The adipogenic and osteogenic differentiation potentials of BMSCs were researched after identification as described previously.

### Plasmid construction and cell transfection

We used the PD-L1 gene sequence NM_001191954.1 in Gene Bank to design and synthesize the PD-L1 siRNA expression cassettes (PD-L1-SECs) according to established protocols. After establishing ligation between the purified siRNA expression cassettes and the psiLentGeneTM vector, the appropriate amount of ligation products were transformed into *E. Coli* DH5α, positive recombinant clones were selected, and the recombinant plasmids were transfected into BMSCs. The BMSCs were inoculated on a 6-well plate one day before transfection, 5 × 10^4^ cells per well, and the complete medium was replaced with 1.9 mL serum-free medium half an hour before transfection. At the time of transfection, the confluence of P2 BMSCs had reached 70–80% per well, was incubated at 37 ℃ for 4–6 h, and then the serum-free medium was replaced with complete medium. After 48 h, PCR and western blot were used to detect the transfection efficiency, and Rat-PD-L1-175 was finally determined as the best PD-L1 siRNA. The gene sequence is 5'-ggaagacaaggaaguuauuca-3' and 5'-Aauaacuuccuugucuuccuu-3'.

### Animals

160–180 g body weight male SD rats were purchased from the experimental animal center of Huazhong University of Science and Technology (HUST; Wuhan, China), and kept under specific pathogen-free (SPF) conditions with food and water provided ad libitum. All the procedures and care of animals were strictly according to the guidelines of the Animal Research Institute Committee of Wuhan Service Technology Co., Ltd., China, and the protocol was approved by the Institutional Animal Care and Use Committee (IACUC) of Wuhan Service Technology Co., Ltd., China.

### Induction of UC model and treatment

After an adaptive feeding of one week, forty male rats were randomly assigned to four groups (n = 10): normal, model, BMSCs control, and PD-L1 siRNA BMSCs. TNBS (Sigma-Aldrich) was used to induce colitis according to Morris et al*.* On the fourth day of model induction, the BMSCs control group and the PD-L1 siRNA BMSCs group were intravenously injected the corresponding BMSCs via the tail vein at a dose of 5 × 10^6^ cells suspended in 1 ml of sterile PBS; the normal group and model group were matched for administered volume. One week after cell injection, all rats were anesthetized and euthanized; their colons were harvested and stored for analysis.

### Evaluation of inflammation

From the first day of modeling, all rats were monitored daily for weight loss, stool quality, and blood in stool to calculate the disease activity index (DAI). After the harvested colons were dissected, part of the colonic tissue was sectioned for hematoxylin–eosin (HE) and the disease scores were generated by histological evaluations performed according to the Modified RILEY Score.

### Western blotting

To test protein content, we performed western blotting as described previously. We used anti-AKT antibody (1:500, Abcam), anti-ser473 antibody (1:5000, Abcam, Cambridge, UK),anti-Thr308 antibody (1:2000, Proteintech), anti-mTOR antibody (1:5000, Abcam), anti-pho-mTOR antibody (1:5000, Abcam), anti-PTEN antibody (1:1000, CST), anti-PD-L1 antibody (1:2000, Proteintech) for primary antibody incubation. β-actin (1:6000, Abcam) was used as a loading control.

### Real-time quantitative PCR (qRT-PCR)

qRT-PCR was used to quantifying the expression of PD-L1 and IL-10 mRNA using a protocol that was described previously. All primer sequences are shown in Table 1. Target gene expression was normalized to β-actin and calculated using the the 2^−ΔΔCt^ method.

**Table 1:** Primer sequences used for polymerase chain reaction

Gene name		Primer sequences (5′to 3′)
PD-L1	Forward	GGAAGACAAGGAAGUUAUUCA
	Reverse	AAUAACUUCCUUGUCUUCCUU
IL-10	Forward	CACTGCTATGTTGCCTGCTCTT
	Reverse	GTCTGGCTGACTGGGAAGTGG
β-actin	Forward	TGCTATGTTGCCCTAGACTTCG
	Reverse	GTTGGCATAGAGGTCTTTACGG

### Flow cytometry

Monocytes were isolated from peripheral blood as described previously. After incubation with anti-CD4 and anti-CD45 (BD Biosciences, San Diego, USA) at 4 ˚C for 30 min in the dark, the cells were stained with anti-FOXP3 (BD Biosciences) and then analyzed by flow cytometry.

### Enzyme-linked immunosorbent assay (ELISA)

To detect the level of IL-10 protein, we collected colon homogenate supernatants for sandwich enzyme-linked immunosorbent assay using a rat IL-10 ELISA kit (NeoBioscience, Shenzhen, China) according to the manufacture’s protocol.

### Statistical analysis

Statistical analysis was performed by using SPSS 22.0 (IBM, Armonk, NY) software. All data are presented as means ± standard deviation (SD). One-way ANOVA or Dunnett’s test (equal variances were not assumed) was used for assessing statistical significance, and P < 0.05 was regarded to indicate a statistically significant difference.

## Results

### Identification of BMSCs

**Fig. 1:**
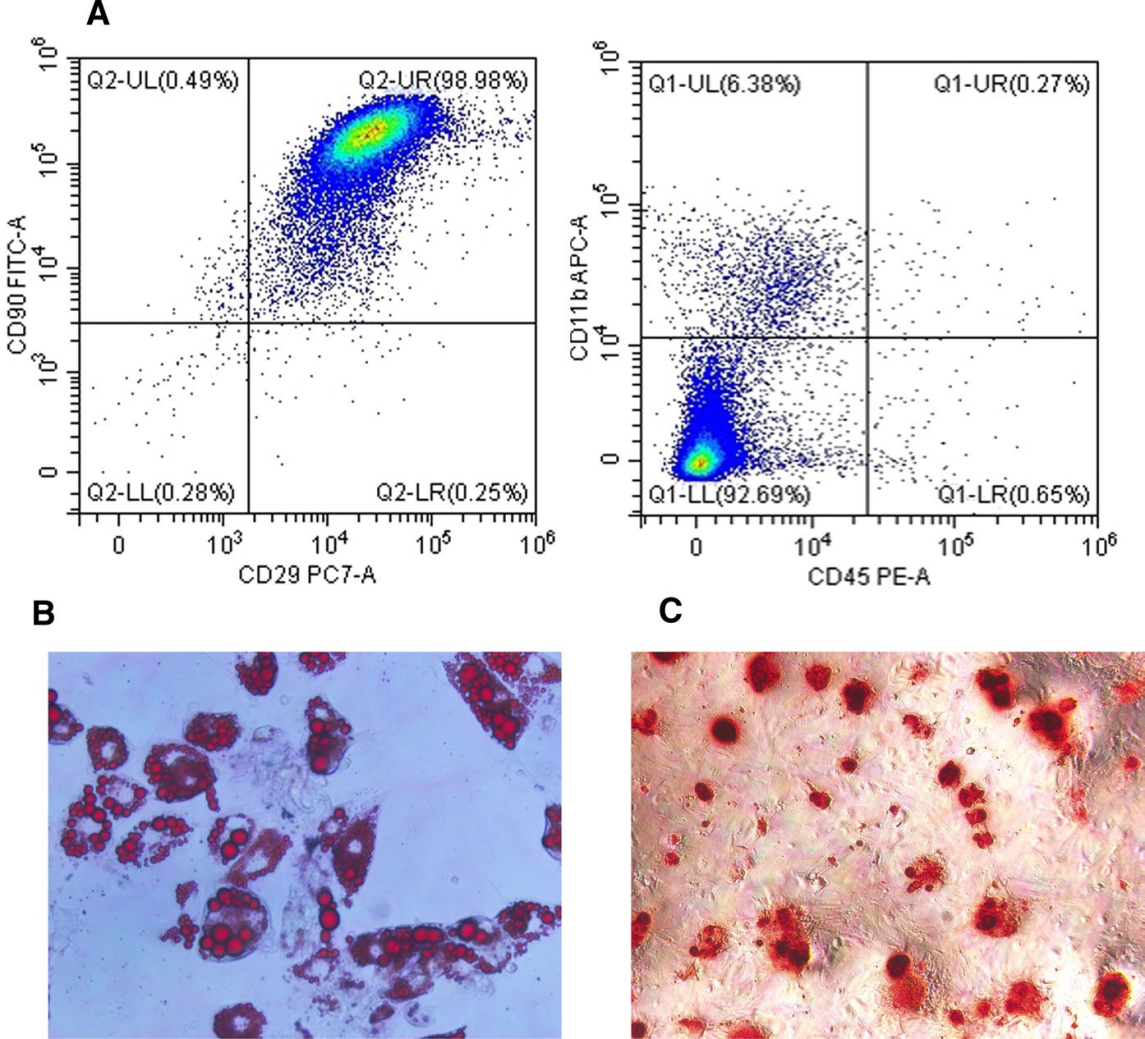
Identification of bone marrow-derived mesenchymal stem cells (BMSCs). **A** Flow cytometric characterization of BMSCs at passage 2. **B** Adipogenic differentiation of BMSCs stained with Oil Red O. Original magnifcation, × 400. **C** Osteogenic differentiation of BMSCs stained with alizarin red. Original magnifcation, × 400

To identify cell purity and differentiation potential, we used a flow cytometry-based cell surface marker strategy and exposed the cells to a specific medium for verifying pluripotency. The flow cytometric analysis revealed that the cells were positive for CD29 and CD90, the surface markers of bone marrow progenitor cells, but negative for CD11b and CD45, the surface markers of hematopoietic cells (Fig. 1A). P2 BMSCs were used for induction. Marked lipid deposition was visible after Oil Red O staining after adipogenic induction (Fig. 1B). Calcium nodules, visible as red spots with alizarin red staining, were observed after osteogenic induction (Fig. 1C).

### PD-L1 siRNA down-regulated PD-L1 expression in BMSCs

**Fig. 2:**
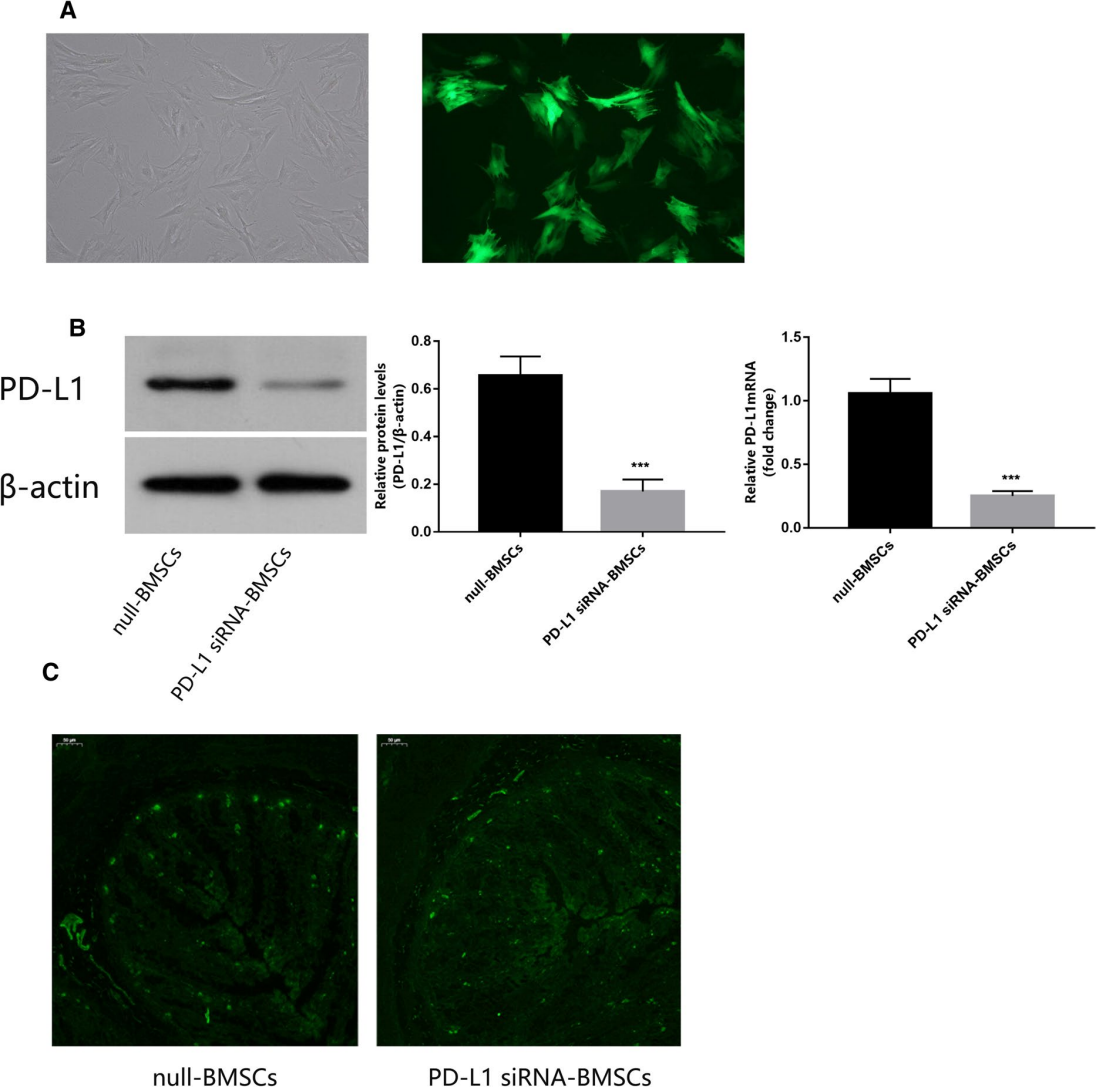
Expression of PD-L1 is down-regulated in PD-L1 siRNA-transfected bone marrow-derived mesenchymal stem cells (BMSCs). **A** The expression of fluorescence-labeled PD-L1 in transfected bone marrow stromal cells was observed under optical and fluorescence microscope. Original magnifcation, × 400. **B** Western blotting analysis of PD-L1 protein in lentivirus-transfected BMSCs and levels of PD-L1 protein and mRNA in lentivirus-transfected BMSCs. **C** GFP-lentivirus transfected BMSCs under fluorescence microscopes. Original magnifcation, × 40. Data are expressed as means ± SD (n = 3). ***P < 0.001 vs. null-BMSCs

To determine the effect of transfection, fluorescent labeling, qRT-PCR, and western blotting of PD-L1 were performed in the PD-L1 siRNA BMSCs and the BMSCs control. The expression of GFP in transfected BMSCs was observed under a fluorescence microscope (Fig. 2A). 72 h after transfection, the levels of PD-L1 mRNA and protein in BMSCs transfected with PD-L1 siRNA were significantly lower than in the control group (Fig. 2B). As such, the plasmid had been successfully transfected and down-regulated PD-L1 as intended. To determine whether the homing ability of BMSCs was different after transfection, the migration efficiency of BMSCs labeled by GFP in colon tissue was observed using an immunofluorescence microscope. The average fluorescence intensity of the PD-L1 siRNA BMSCs group was 16.346, and that of the null-BMSCs group was 16.26, calculated by Image J. This showed that there was no significant difference in the number of BMSCs colonized in the intestine between the two groups and that the transfection had no effect on the homing of BMSCs (Fig. 2C).

### Down-regulation of PD-L1 inhibited remission of TNBS-induced colitis by BMSCs

**Fig. 3:**
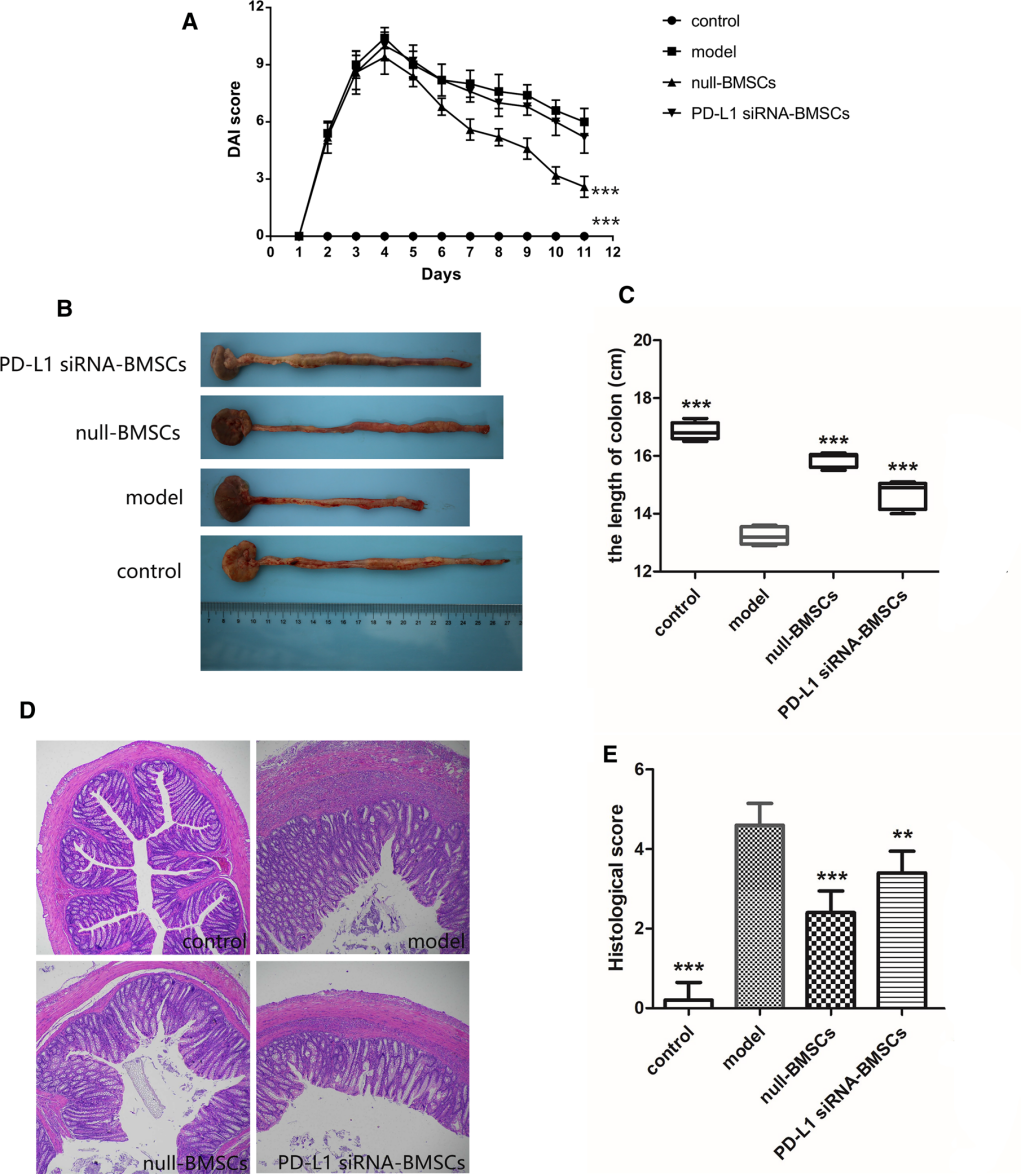
Expression of PD-L1 enhances the protective effects of bone marrow-derived mesenchymal stem cells (BMSCs) in rats with 2,4,6-trinitrobenzene sulfonic acid (TNBS)-induced colitis. **A** The disease activity index (DAI) of rats was monitored daily. **B** Colons of rats from different treatments are shown. **C** Colonic length of rats under different treatments. **D** Colon specimens stained with hematoxylin and eosin were analyzed. Original magnification, × 40. **E** Colonic histological scores.Data are expressed as means ± SD (n = 5).**P < 0.01, ***P < 0.001 vs. model group

To evaluate the effect of PD-L1 in TNBS-induced colitis of rats, we monitored weight loss and calculated DAI daily from modeling. Rats in the model group showed the most marked weight loss and the highest DAI of all groups. For the cell therapy groups, weight loss and DAI decreased significantly in the BMSC group compared to the PD-L1 siRNA BMSCs group (Fig. 3A). Colonic inflammation is known to result in colon shortening. Compared with the control group, colon lengths were shortened by 21.47% in the model group, 5.93% in the null-BMSCs group, and 13.05% in the PD-L1 siRNA BMSCs group (Fig. 3B, C); the difference between the two cell therapy groups was significant. Histological analysis showed mucosal erosion and extensive inflammatory cellular infiltration the colons of the model group; cell therapy mitigated this damage and reduced the histological score. However, the BMSC control group showed less inflammation than the PD-L1 siRNA BMSCs group (Fig. 3D, E). Taken together, we show that BMSCs inhibited intestinal inflammation in TNBS-induced rats via PD-L1.

### Down-regulation of PD-L1 in BMSCs decreased Tregs in TNBS-induced colitis

**Fig. 4:**
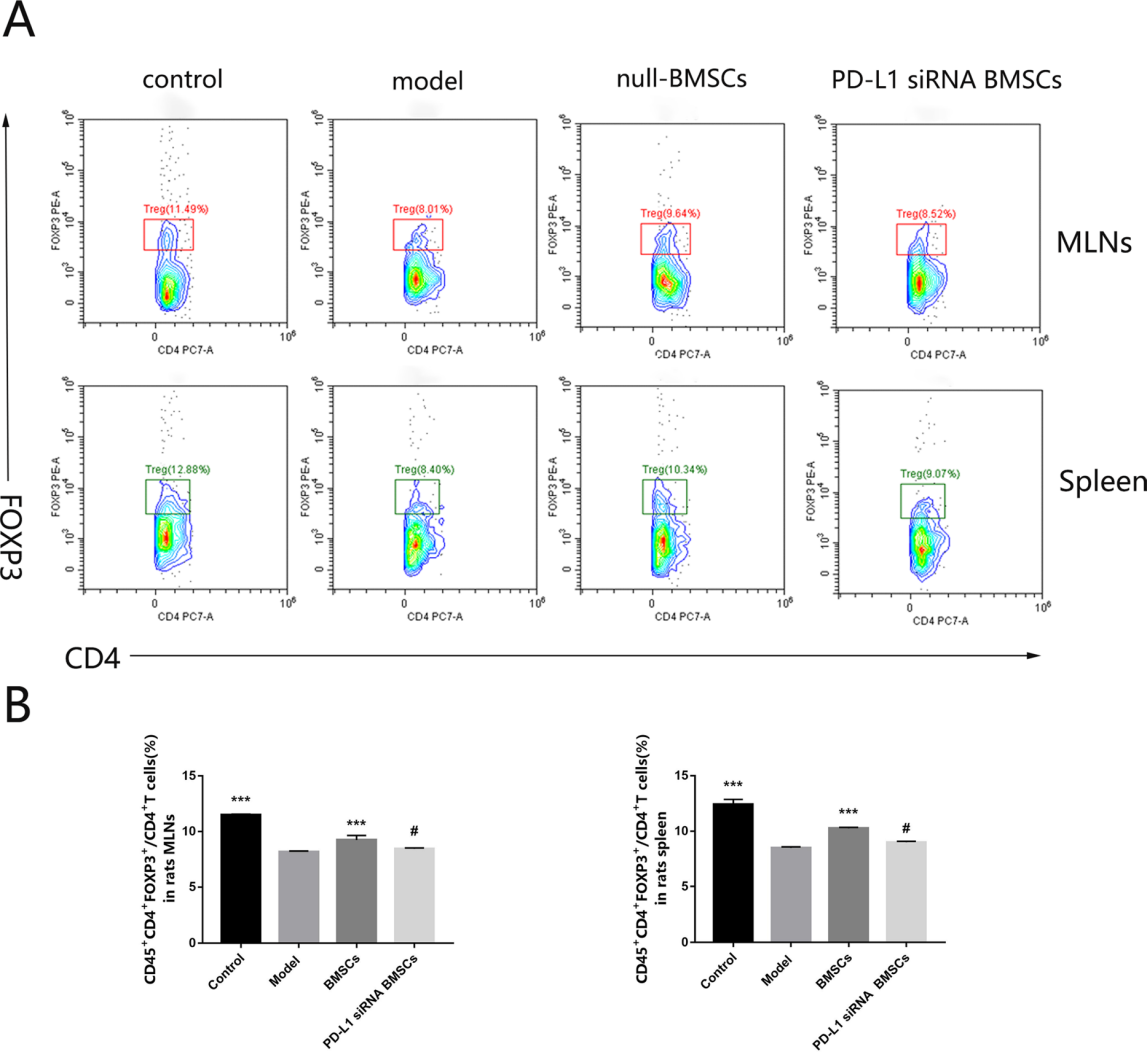
Expression of PD-L1 up-regulates Tregs cells in rats with 2,4,6-trinitrobenzene sulfonic acid (TNBS)-induced colitis. **A** Flow cytometry analyzed the frequencies of Tregs in mesenteric lymph nodes (MLNs) and spleen. **B** Average frequencies of Tregs in MLNs and spleen. Data are expressed as means ± SD (n = 3). #P > 0.05, ***P < 0.001 vs. model group

**Fig. 5:**
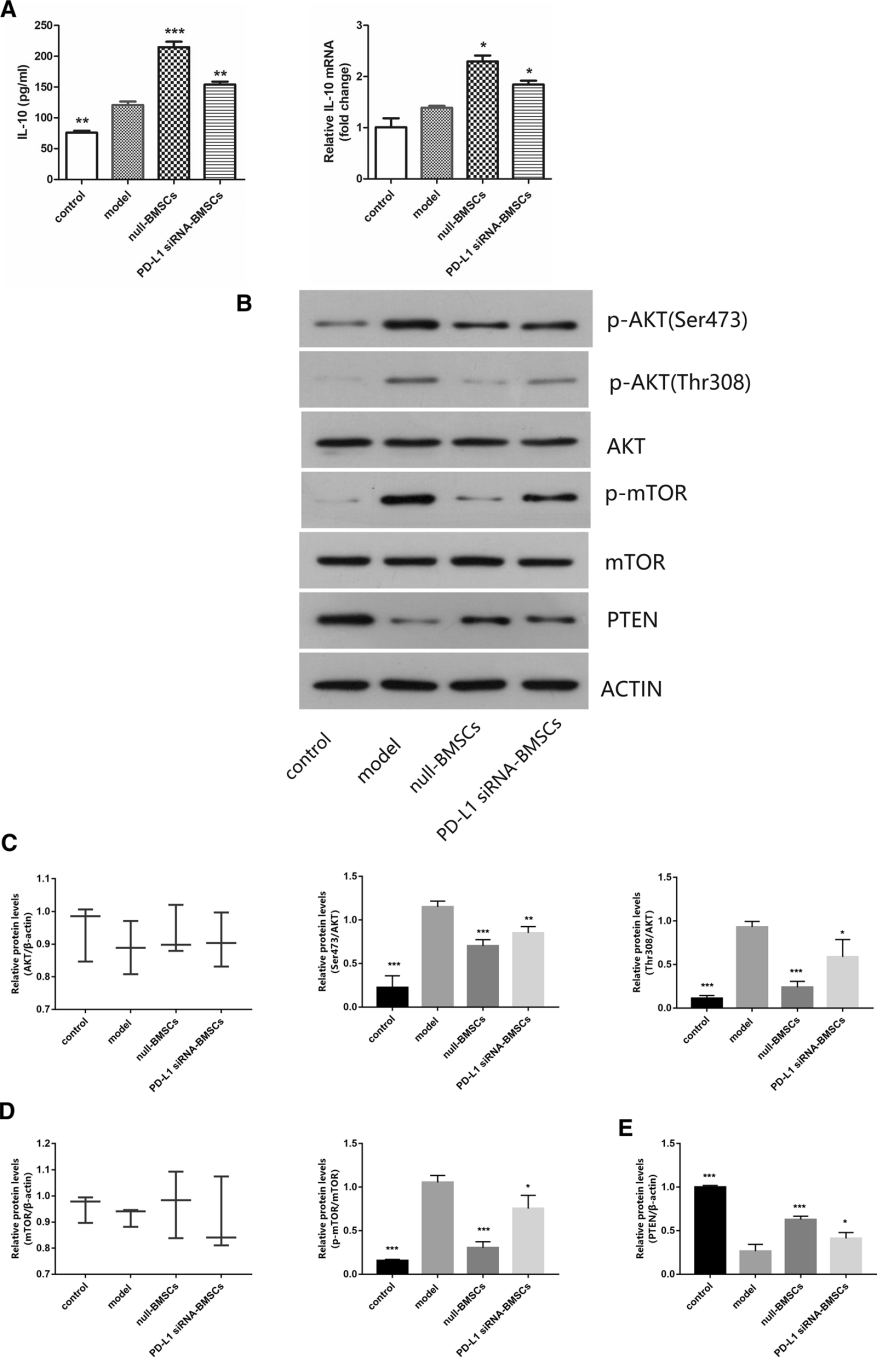
**A** Levels of IL-10 in colon tissues of each group were measured by enzyme-linked immunosorbent assay (Elisa) and quantitative real-time polymerase chain reaction (qRT-PCR) analyses of IL-10 mRNA expression in colons. **B** Western blotting analysis of phosphor-Akt (Ser473),phosphor-Akt (Thr308),AKT, phosphor-mTOR,mTOR and PTEN protein. **C** Levels of Ser473 protein and Thr308 protein in colon tissues of each group. **D** Levels of phosphor-mTOR protein in colon tissues of each group. **E** Levels of PTEN protein in colon tissues of each group. Data are expressed as means ± SD (n = 3). *P < 0.05,**P < 0.01, ***P < 0.001 vs. model group

To investigate whether BMSCs promote Treg differentiation via PD-L1, we analyzed the percentage of Tregs in lymphocytes of spleen and mesenteric lymph nodes. As anti-inflammatory cells, Tregs are reduced in the rat model of UC induced by TNBS, and BMSCs transplantation can promote Tregs in UC rats according to our previous studies (Nan et al. 2018). Our present results showed that we reproduced the results of our previous experiments and that PD-L1 inhibition in BMSCs decreased Tregs compared with the BMSCs control (Fig. 4A, B). In addition, the PD-L1 siRNA BMSCs showed down-regulated IL10, the signature Treg cytokine, by PCR and ELISA compared with the BMSC controls (Fig. 5A). In general, PD-L1 exerts an important role in promoting Treg differentiation by BMSCs.

### Down-regulation of PD-L1 inhibited the Akt/mTOR pathway

The Akt/mTOR pathway is one of the PI3K down-stream pathways and participates in regulating the differentiation of naive T cells, which differentiate into Tregs through an inhibition of the Akt/mTOR pathway. To test whether PD-L1 on BMSCs promotes Tregs and relieves inflammation via the Akt/mTOR pathway, western blotting of Akt phosphorylation and mTOR phosphorylation were performed. We found that phosphor-Akt (Ser473), phosphor-Akt (Thr308), and phosphor-mTOR were increased in the PD-L1 siRNA BMSC group compared with the BMSC control group (Fig. 5B, C, D). We also found that PTEN, which is important for antagonizing PI3K signaling, was increased (Fig. 5E) As such, we found that BMSCs induced Tregs by activating the Akt/mTOR pathway via PD-L1 in TNBS-treated rats.

## Discussion

UC and Crohn’s disease (CD) belongs to the family of IBD. The typical pathologic manifestations of UC are mucosal ulceration, submucosal edema, lymphocyte infiltration, and fibrosis; these features are related to a T cell-mediated immune dysregulation that remains incompletely understood (Gui et al. 2018; Schardey et al. 2019). Many animal models are utilized to interrogate UC. In this study, we utilized the TNBS approach to induce colitis in rats, which mimics human UC in terms of symptoms and pathological processes.

In recent years, mesenchymal stem cell (MSC)-based therapy has attracted significant attention owing to the immunoregulatory capacity and multi-lineage differentiation ability of MSCs. Currently, the application of MSC therapy has focused on local or systematic transplantation, combined with genetic modification and tissue engineering (Allbright et al. 2018; Khayambashi et al. 2021; Lin et al. 2019). BMSCs, an important subset of MSCs, have shown significant promise in the treatment of IBD (Nan et al. 2018; Zuo et al. 2015). The gut, the site of IBD pathology, is itself a unique immune organ and is damaged when the balance between pro-inflammatory and anti-inflammatory cells is disrupted (Chen et al. 2020; Wang et al. 2019). Some pro-inflammatory cells such as Th1 and Th17 T cells, differentiated from naïve T cells, can induce and maintain intestinal inflammation by secreting effector cytokines, while Tregs, also derived from naive T cells, are important complementary cells that suppress their pro-inflammatory counterparts (Hu et al. 2021; Imam et al. 2018; Yang et al. 2020). Extensive evidence shows that Tregs are decreased in UC and that promoting Treg differentiation alleviates colitis in animal models (Nan et al. 2018; Xu et al. 2019; Zhang et al. 2020). Thus, BMSCs and Tregs offer much therapeutic promise. Casiraghi et al*.* (Casiraghi et al. 2008) reported that transplantation of MSCs induces Tregs in vivo, and our preliminary experiment showed that the percentage of Tregs is increased after the administration of BMSCs in TNBS-induced colitis (Nan et al. 2018). However, some have found that transplantation of MSCs can enhance tumor growth in some animal models which is thought to be related to the multipotency of these cells (Biswas et al. 2019; Dong et al. 2018). So to exploit these findings in therapeutic trials, we first need to gain a comprehensive understanding of the complex interaction between MSCs and Tregs; this study was aimed at clarifying some of the regulatory mechanisms between these two cell types, based on our previous findings.

Recently, there has been great interest in the functions of PD-1 and its ligand PD-L1 in regulating immunological tolerance and autoimmunity (Sun et al. 2018). Several studies have demonstrated that the PD-1/PD-L1 pathway exerts pivotal roles in physiological and pathological processes including activation and differentiation of T cells, oncogenesis, and some chronic inflammation (Dammeijer et al. 2020; Gao et al. 2019; Weyand et al. 2018). Both are expressed on T cells, B cells, macrophages, and some dendritic cells (DCs). Meanwhile, PD-L1 is constitutively expressed on MSCs in mice (Davies et al. 2017a, b; Jin et al. 2011). As such, BMSCs might promote the activation of naïve T cells and induce their differentiation into Tregs via the PD-1/PD-L1 pathway. In T cell differentation, it is the interaction between PD-1 and PD-L1 that drives the generation of Tregs (Francisco et al. 2009). Furthermore, research indicates that Akt signaling, which is necessary for the activation and proliferation of naïve T cells, is dependable for the development and function of Tregs (Abdullah et al. 2021; Han et al. 2021; Jones et al. 2019). During the activation and proliferation of T cells, binding of PD-1 on the surface of naive T cells and PD-L1 leads to the phosphorylation of ITIM and ITSM on the cytoplasmic domain and the recruitment of SHP-1 and SHP-2. SHP-1 and SHP-2 inhibit the activation of PI3K and prevent the phosphorylation of Akt, down-stream of PI3K (Jin et al. 2011), and then suppress the Akt/mTOR signaling cascade, consequently modulating the “molecular switch” in naiïve T cells to preferentially induce the development of Tregs (Hawse et al. 2017). Simultaneously, the inhibition of PI3K blocks the proliferation and survival of T cells, which maintains the function of Tregs (Sefik et al. 2015). In this study, we showed that BMSC administration inhibited the Akt/mTOR pathway, up-regulated the expression of PTEN, the inhibitory signal of PI3K, and raised the percentage of the Treg population, as well as their cytokine IL-10. The PD-L1 siRNA BMSCs group showed the opposite phenotype.

In conclusion, BMSCs improve TNBS-induced colitis by expressing PD-L1, and its mechanism is mainly related to the inhibition of the Akt/mTOR pathway which can induce Treg differentiation. PD-L1 is an important target of BMSCs in the treatment of UC.

## Data Availability

The datasets generated during and/or analyzed during the current study are available from the corresponding author on request.
